# Testicular ultrastructure and hormonal changes following administration of tenofovir disoproxil fumarate-loaded silver nanoparticle in type-2 diabetic rats

**DOI:** 10.1038/s41598-022-13321-y

**Published:** 2022-06-10

**Authors:** Samuel Oluwaseun Olojede, Sodiq Kolawole Lawal, Oluwaseun Samuel Faborode, Ayobami Dare, Okikioluwa Stephen Aladeyelu, Roshila Moodley, Carmen Olivia Rennie, Edwin Coleridge Naidu, Onyemaechi Okpara Azu

**Affiliations:** 1 Discipline of Clinical Anatomy, School of Laboratory Medicine & Medical Sciences, Nelson R Mandela School of Medicine, University of KwaZulu-Natal, 719 Umbilo Road, Durban, South Africa; 2 Discipline of Physiology, School of Laboratory Medicine & Medical Sciences, College of Health Sciences, Westville Campus, University of KwaZulu-Natal, Durban, South Africa; 3 Department of Physiology, Faculty of Basic Medical Sciences, Bingham University, Karu, Nasarawa State, Nigeria; 4 The Department of Chemistry, The University of Manchester, Manchester, UK; 5 Department of Human, Biological & Translational Medical Sciences, School of Medicine, University of Namibia, Hage Geingob Campus, Private Bag 13301, Windhoek, Namibia

**Keywords:** Cell biology, Chemical biology, Drug discovery, Physiology, Endocrinology, Nanoscience and technology

## Abstract

Reproductive dysfunctions (RDs) characterized by impairment in testicular parameters, and metabolic disorders such as insulin resistance and type 2 diabetes mellitus (T2DM) are on the rise among human immunodeficiency virus (HIV) patients under tenofovir disoproxil fumarate (TDF) and highly active antiretroviral therapy (HAART). These adverse effects require a nanoparticle delivery system to circumvent biological barriers and ensure adequate ARVDs to viral reservoir sites like testis. This study aimed to investigate the effect of TDF-loaded silver nanoparticles (AgNPs), TDF-AgNPs on sperm quality, hormonal profile, insulin-like growth factor 1 (IGF-1), and testicular ultrastructure in diabetic rats, a result of which could cater for the neglected reproductive and metabolic dysfunctions in HIV therapeutic modality. Thirty-six adult Sprague–Dawley rats were assigned to diabetic and non-diabetic (n = 18). T2DM was induced by fructose-streptozotocin (Frt-STZ) rat model. Subsequently, the rats in both groups were subdivided into three groups each (n = 6) and administered distilled water, TDF, and TDF-AgNP. In this study, administration of TDF-AgNP to diabetic rats significantly reduced (p < 0.05) blood glucose level (268.7 ± 10.8 mg/dL) from 429 ± 16.9 mg/dL in diabetic control and prevented a drastic reduction in sperm count and viability. More so, TDF-AgNP significantly increased (p < 0.05) Gonadotropin-Releasing Hormone (1114.3 ± 112.6 µg), Follicle Stimulating Hormone (13.2 ± 1.5 IU/L), Luteinizing Hormone (140.7 ± 15.2 IU/L), testosterone (0.2 ± 0.02 ng/L), and IGF-1 (1564.0 ± 81.6 ng/mL) compared to their respective diabetic controls (383.4 ± 63.3, 6.1 ± 1.2, 76.1 ± 9.1, 0.1 ± 0.01, 769.4 ± 83.7). Also, TDF-AgNP treated diabetic rats presented an improved testicular architecture marked with the thickened basement membrane, degenerated Sertoli cells, spermatogenic cells, and axoneme. This study has demonstrated that administration of TDF-AgNPs restored the function of hypothalamic-pituitary–gonadal axis, normalized the hormonal profile, enhanced testicular function and structure to alleviate reproductive dysfunctions in diabetic rats. This is the first study to conjugate TDF with AgNPs and examined its effects on reproductive indices, local gonadal factor and testicular ultrastructure in male diabetic rats with the potential to cater for neglected reproductive dysfunction in HIV therapeutic modality.

## Introduction

There has been a neglected aspect of reproductive dysfunction in human immunodeficiency virus 1 (HIV-1) infected persons, with increasing reports of reproductive dysfunctions (RDs) in these people under antiretroviral drugs^1^. Although RDs are part of HIV complications widely reported in the literature before introducing highly active antiretroviral therapy (HAART)^2,3^. Nevertheless, the roll-out of HAART since the mid-80s and to date, antiretroviral therapy is now perceived as a direct or indirect cause of RDs via various mechanisms of action^4,5^. Among several adverse effects of HAART are insulin resistance and type 2 diabetes mellitus (T2DM), which were documented as HIV-1 therapeutic detrimental effects, especially HAART^6^.

There is now an increase in the cases of T2DM among HIV-infected persons with continuing exposure to HAART^7^. A recent study has shown that most approved HAART, especially the fixed-dose combination with tenofovir, lamivudine, or efavirenz, is associated with diabetes mellitus (DM)^8^. Nonetheless, the possible development of DM and subsequent RDs associated with monotherapy of tenofovir disoproxil fumarate (TDF), the backbone of the HAART regimen, has not been widely investigated. Imperatively, the mechanism underlying the metabolic and RDs pathogenesis in HAART has been described. Several studies have delineated leptin and adiponectin as crucial biomarkers and regulators of energy metabolism with vital roles in modulating the immunological, neuronal, endocrine, and metabolic functions^9–11^. The primary source of energy metabolism in the testis remains the Sertoli cells. The Sertoli cells function to absorb glucose in the plasma and convert it to lactate and supply the cells inside the blood-testis barrier in the form of energy^12^. Therefore, alteration in energy metabolism or energy imbalance due to reduction in the levels of these markers of energy metabolism has been attributed to the cause of metabolic disorders and RDs in the HAART era^13,14^.

To further understand the interactions of antiretroviral therapy and subsequent development of reproductive or endocrine dysfunction, it is necessary to explore the role of local gonadal factors and the reproductive hormones. This is because local gonadal factors like insulin growth-like factor 1 (IGF-1) also play a significant role in developing and maintaining reproductive functions^15^. In addition to luteinizing hormone (LH) and follicle-stimulating hormone (FSH), research evidence has pointed at IGF-1 as a testicular modulator protein/local gonadal factor for controlling the testicular functions and integrity^16,17^. Also, a normal level of IGF-1 has been attributed to enhanced glucose tolerance, improved insulin sensitivity, excellent glycaemic control, increased appetite and anti-inflammatory activity^18,19^.

However, the long-term administration and adverse effects of TDF on renal functions and parameters have been extensively explored^20–22^. Previously, the implication of DM on male reproductive parameters such as mtDNA damage and increased sperm nuclear damage with subsequent impairment on the reproductive ability was reported^23^. In addition, azoospermia, significantly reduced sperm concentration^24,25^ and decreased sperm motility^26^ were all documented in the literature. With this evidence of reproductive derangements in HAART regimen and impairment in reproductive parameters in DM, there is a triple burden of diseases; HIV-1 infection, T2DM and RDs. Awodele, Popoola^26^ revealed that administration of either fixed-dose combination or monotherapy of antiretroviral drugs has possible reproductive adverse effects.

In recent times, novel HIV therapeutic agents have been on the rise. Nevertheless, a remarkable feat has been achieved in employing nanoparticles to deliver these drugs to optimize the efficacy of the currently administered antiretroviral agents and proffer solutions to the various adverse effects. In this regard, different nanoparticles (NPs) have been successfully synthesized to load TDF. Chitosan-based nanoparticles coupled with TDF was prepared, characterized and used for optimal oral absorption in experimental rats^27^. Also, Pokharkar and Co-workers fabricated polymer-lipid nanocarrier to deliver TDF intranasally^28^. In another attempt, TDF loaded poly(lactic-co-glycolic acid) (PLGA)/stearylamine (SA) was synthesis as a new vaginal microbicide delivery vehicle^29^.

Despite these tremendous efforts, the characteristic large surface area to volume ratio, virucidal activity, and targeted delivery ability of the silver nanoparticles (AgNPs) are yet to be exploited. More so, the increase in reproductive desires in individuals living with HIV-1 infection thereby necessitates the conception of this study. A study aimed to investigate the effects of TDF loaded AgNPs on the testicular parameters, hormonal profile, IGF-1 and testicular ultrastructure of the diabetic rat model. This may cater for the neglected metabolic and reproductive adverse effects in managing HIV infection.

## Materials and methods

### Ethical statement

Before the commencement of this research, ethical approval was sought and received from the Animal and Research Ethics Committee (AREC) at the University of KwaZulu-Natal (UKZN) with the approval number AREC/043/019D. This study was carried out at Biomedical Resource Unit (BRU), UKZN, Westville Campus, under the Principle of Laboratory Animal Care of the UKZN approved guidelines and principle of the 3Rs. Also, this study follows the recommendations in the Animal Research: Reporting of In Vivo Experiments (ARRIVE) guidelines, and the animals received humane care.

### Chemical and drug

Tenofovir disoproxil fumarate (TDF), a drug from the nucleoside reverse transcriptase inhibitors (NRTI) classes of antiretroviral drugs with a human equivalent dose of 300 mg, were purchased from Dis-Chem Pharmacy, Ballito, South Africa. Fructose (Frt), streptozotocin (STZ), trisodium citrate (TSC), sodium hydroxide (NaOH), and silver nitrate (AgNO_3_) of analytical grade were sourced from Sigma-Aldrich Company, Johannesburg, South Africa.

### Synthesis of AgNP

This study used AgNO_3_ as a precursor and TSC to reduce and stabilize the synthesized AgNPs^30^. Exactly, 0.03 mol/dm^3^ were thoroughly mixed with aqueous 0.1 mol/dm^3^ TSC, and the resultant solution was under continuous stirring for 5 min at 90 °C, and NaOH (pH 10.5) was used to change the pH for 90 min. A colour change from colourless to amber yellow was observed. The synthesized AgNP was cool at room temperature, centrifuged at 12 000 rpm for 15 min, and oven-dried at 40 °C overnight^31^. Thereafter, the 0.35 M of TDF was mixed with 100 mL of synthesized AgNP, and the mixture was under continuous stirring in ultra-sonication to ensure proper reaction of the components (TDF and AgNPs).

TDF-AgNP was centrifuged at 4500 rpm, for 40 min at 40 °C to discrete the unincorporated drug. The supernatant gotten was analyzed using a UV spectrophotometer at a wavelength of 274 nm to calculate the quantity of unincorporated drug (W1) from the total amount of drug coupled with silver nanoparticle (W2). The percentage of drug incorporation efficiency (% IE) was estimated by employing the below equation^32^.

1 $$\text{Percentage incorporated efficiency }(\% \text{IE}): =\frac{\text{TW}2-\text{TW}1}{\text{TW}2}\times 100\%$$

TW2 is the total amount of TDF loaded with silver nanoparticles, and TW1 represents the quantity of unincorporated TDF. The synthesized AgNP and conjugation of TDF-AgNP were confirmed using ultraviolet–visible (UV–Vis) spectroscopy (Shimadzu MultSpec-1501, Shimadzu Corporation, Tokyo, Japan). Also, the Fourier transform infrared (FTIR) spectroscopy (Perkin-Elmer Universal ATR spectrometer, USA) confirmed the functional groups and bonds. The morphology and size of the synthesized nanoparticles were examined by a high-resolution transmission electron microscope (HR-TEM, JEOL 2100, Japan) operated at a voltage of 200 kV and a field emission scanning electron microscope (FESEM, Carl Zeiss, Germany) performed at a voltage of 5 kV. Also, the elemental constituents were confirmed by energy dispersive X-ray (EDX, Aztec Analysis Software, England).

### Experimental design

A total of thirty-six (n = 36) adult male Sprague–Dawley rats weighing between 230–250 g was used for this research. The rats were housed in well-ventilated plastic cages (3 rats per cage having dimensions of 52 cm × 36 cm and 24 cm, length, width and high, respectively, with bedding of softwood shavings). The rats were maintained under standardized animal house conditions with temperature ranges from 23 to 25 °C; 12 h of natural light per day. The rats were fed with standard rat pellets from Meadow feeds, Division of Astral Operations Limited, Durban, South Africa, and water was given ad libitum.

Following the two weeks of acclimatization, the initial body weights of the rats were taken recorded, and then the rats were randomly assigned into diabetic (n = 18) and non-diabetic n = 18) groups. The rats allotted to the diabetic group received fructose (Frt) and streptozotocin (STZ) to induce T2DM, following the protocol previously described by Wilson and Islam^33^. Based on this model, 10% frt was dissolved in drinking water ad-libitum for two weeks to cause insulin resistance. After that, the rats were induced with 40 mg/kg of STZ freshly prepared in 0.1 M citrate buffer (pH 4.5) based on body weight. More so, the rats in the non-diabetic group received the same volume of 0.1 M citrate buffer (pH 4.5) intraperitoneally. The diabetic model was characterized by checking hyperglycaemia through fasting blood glucose using glucometer and strips (Acucheck^(R)^, Boehringer-Mannheim, Germany). The experimental rats with a blood glucose level of 200 mg/dL and above were considered diabetic and included in the diabetic group. The diabetic and non-diabetic groups were further divided into three groups each (n = 6) as follows.

Group I represent non-diabetic control rats (NDC)-received 1 mL distilled water daily (p.o).

Group II indicates non-diabetic tenofovir rats (NDT)-were treated with 26.8 mg/kg/body weight daily of tenofovir (p.o).

Group III represents non-diabetic TDF-AgNP rats (NDNT)-were administered 6.7 mg/kg/body weight TDF-AgNP (daily i.p).

Group IV represents diabetic control rats (DC)-received 1 mL distilled water daily (p.o).

Group V indicates diabetic tenofovir rats (DT)- were treated with 26.8 mg/kg/body weight daily tenofovir (p.o).

Group VI represent diabetic TDF-AgNP rats (DNT)-were administered 6.7 mg/ml/body weight TDF-AgNP daily (i.p).

### Animal treatment

The tablet form of TDF (300 mg) was used in this experiment. The tablets were squashed into powder form, and adequate amounts weighed out following the recommended human doses. The animal dose was calculated from the human equivalent dose (HED) using the recommended equation by the United States Food and Drug Administration (FDA)^34,35^;

$${\text{HED}}\left(\frac{{\text{mg}}}{{\text{kg}}}\right)=\frac{{\text{Animal NOAEL}}\left(\frac{{\text{mg}}}{{\text{kg}}}\right) \times {\text{Animal Weight}}\left({\text{kg}}\right)}{{\text{Human weight }}\left({\text{kg}}\right)^{1-0.67}}.$$

The rats in treatment groups were administered 26.8 mg/kg/day of TDF based on this formula.

The experiment ran for thirteen weeks, while the administration ran for 56 days. More so, thorough environmental enrichment practices and application of 3Rs and five freedoms for animal welfare pain, discomfort and distress were considered throughout the experiment. Following the application of 3R's, daily administration in groups III and VI through intraperitoneal were reduced to 6 days a week. The animals were euthanized by excessive isoflurane inhalation 24 h after the last administration on day 57. The blood samples were collected via intracardiac puncture into different sample bottles, and testes were excised for various analyses.

### Determination of sperm count and viability

The sperm count and viability were analyzed as previously described^36^. Shortly after the euthanization, the cauda epididymides of the experimental rats were removed and collected in 5 mL of normal saline in each labelled bottle. A 2 mL was withdrawn from it and used to mince 1 cauda epididymis in the petri dish. This dilution factor (2 mL) was kept constant throughout and for all the rats. After that, the epididymal fluid was thoroughly mixed, and approximately 20 μl of this diluted specimen was transferred to each of the counting chambers of the hemocytometer and counted with Bio-Rad^R^ equipment (TC20 Machine). Both sides of the counting chamber were used for each specimen, and the average was recorded to the nearest millions /millilitre^37^. Sperm viability was assessed by staining a dry spermatozoa smear on a glass slide with eosin-nigrosine staining following observation under a light microscope (Leica DM 500) at 400× magnification.

### Analysis of reproductive hormones

Blood samples for the hormonal assay were collected through cardiac puncture after the animals were euthanized. Blood samples were transferred into serum bottles, allowed to stand for 30 min and centrifuged at 3000 rpm for 15 min in a Beckman bench centrifuge. According to the manufacturer's instructions, the serum concentration of reproductive hormones was determined using enzyme-linked immunosorbent assay (ELISA) kits (Elabscience). The serum levels of Gonadotropin-releasing hormone (GnRH), follicle-stimulating hormone (FSH), Luteinizing hormone (LH) and testosterone were determined by using ELISA kits (Elabscience) rat specific kits with catalogue numbers: E-EL-R0597, E-EL-R0391, E-EL-R0026 and E-EL-R0389 for GnRH, FSH, LH and testosterone, respectively, by manufacturer's protocols. The analyses of the samples were carried out in duplicate, and the values were estimated from the standard curve produced from the calculated concentrations.

### Determination of Insulin-like growth factor 1 (IGF-1) expression

An equal amount of 0.5 g of each harvested testes was homogenized using 5 mL sodium phosphate buffer solution with 1% Triton X-100 (50 mM; pH 7.5). The testicular homogenates were centrifuged at 20,000 g in a Centrikon H-401 (Germany) centrifuge for 10 min at a temperature of 4 °C. After that, the supernatants were collected, decanted into 2 mL Eppendorf tubes, labelled and preserved at − 80 °C until further analysis. The testis homogenates of IGF-1 level was determined using double-antibody sandwich enzyme linked-immunosorbent assay (ELISA) kit (Elabscience) with catalogue number E-EL-R0010, Lot: E3YXQMELZQ following the manufacturer's protocol.

### Electron microscopy procedure

Testis was cut into small 1 mm^3^ sections and instantly fixed in modified Karnovsky's fixative containing 0.2 M cacodylate buffer, 0.2% picric acid, 4% paraformaldehyde, 0.02% calcium chloride, and 4% glutaraldehyde. After that, the tissues were dehydrated in graded acetone solutions and embedded in resin (Araldite). The KOS microwave tissue processor was used for the post embedding procedures such as rinsing, post-fixation, dehydration and infiltration. Also, semi-thin pieces were cut at a thickness of 1 μm with a glass knife using Leica Ultracut R Ultramicrotome. The tissues were stained with 1% toluidine blue dissolved in 1% borax for about 30 min at a temperature of 40–50 °C. After that, the stained tissues were washed, dried and examined with the aid of the light microscope for general orientation. The Leica Ultracut R Ultramicrotome (Leica Microsystems, Milton Keynes, England) was used to obtain tissue sections with thickness less than 100 nm. The grids were then stained with 2% alcoholic uranyl acetate for 10 min in the dark, thoroughly washed in milliq water, and air dry before the examination. The sections were then viewed and photographed using the FEI TECNAI™ Transmission Electron microscope (TEM) (120kv)^38^. Furthermore, the TEM was carried out at the Microscopy and Microanalysis Unit (MMU), University of KwaZulu-Natal, Westville, South Africa.

### Statistical analysis

In this study, the statistical tool used was one-way analysis of variance (ANOVA) to determine the mean differences between the groups. Also, Tukey's multiple comparisons posthoc tests were performed using Graph pad prism®, statistical software version 7.0. The results were visually displayed and expressed as mean ± standard error of the mean (SEM) at a 95% confidence level (*P* < 0.05).

### Ethical approval and consent to participate

The ethical permission for this study was obtained from the Animal and Research Ethics Committee (AREC) of the University of KwaZulu-Natal with the approval number AREC/043/019D. Also, this study was conducted at the Biomedical Resource Unit (BRU) of the University of KwaZulu-Natal, Westville Campus, under the Principle of Laboratory Animal Care of the University of KwaZulu-Natal standard approved guidelines.

## Results

### Characterization of AgNPs and TDF-AgNPs

The transmission and scanning electron microscopy revealed the sizes of spherical nanoparticles ranging from 12 to 22 nm. The UV–vis spectroscopy showed an absorption peak between 325–328 nm for the nanoconjugates. More so, the presence of the functional groups (O–H, C–N) in the nanoconjugates was revealed by Fourier-transform infrared spectroscopy. Also, elemental constituents such as carbon, oxygen, silver, sodium, copper, and phosphorus were revealed by Energy-dispersive X-ray spectroscopy.

### TDF-AgNP and blood glucose level

**Figure 1:**
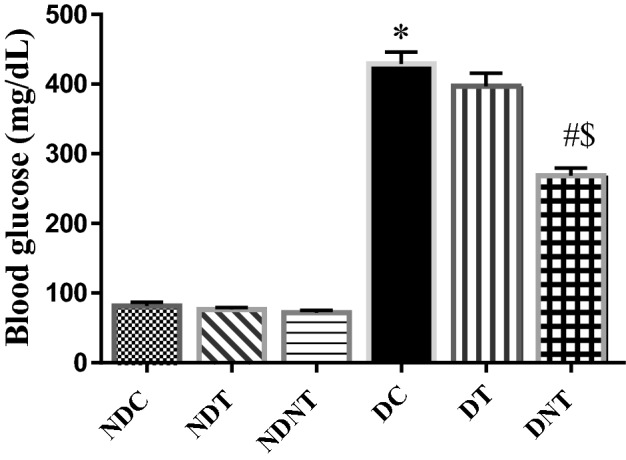
This figure illustrates the blood glucose level of the treated groups and control. As shown in Fig. 1., the blood glucose level in diabetic control rats were significantly increased compared with non-diabetic control. Also, administration of TDF-AgNP to diabetic rats significantly reduced the blood glucose levels compared with diabetic control rats and rats treated with TDF.

Figure 1 shows a significant increase (p < 0.05) in blood glucose levels of frt-STZ induced diabetic control (DC) rats compared with non-diabetic control (NDC) rats. The blood glucose levels in diabetic rats treated with TDF-AgNP were significantly decreased (p < 0.05) compared with diabetic control rats and diabetic rats treated with TDF. More so, no significant difference in the blood glucose levels of non-diabetic rats administered TDF-AgNP and TDF.

### TDF-AgNP, sperm count and sperm viability

**Figure 2:**
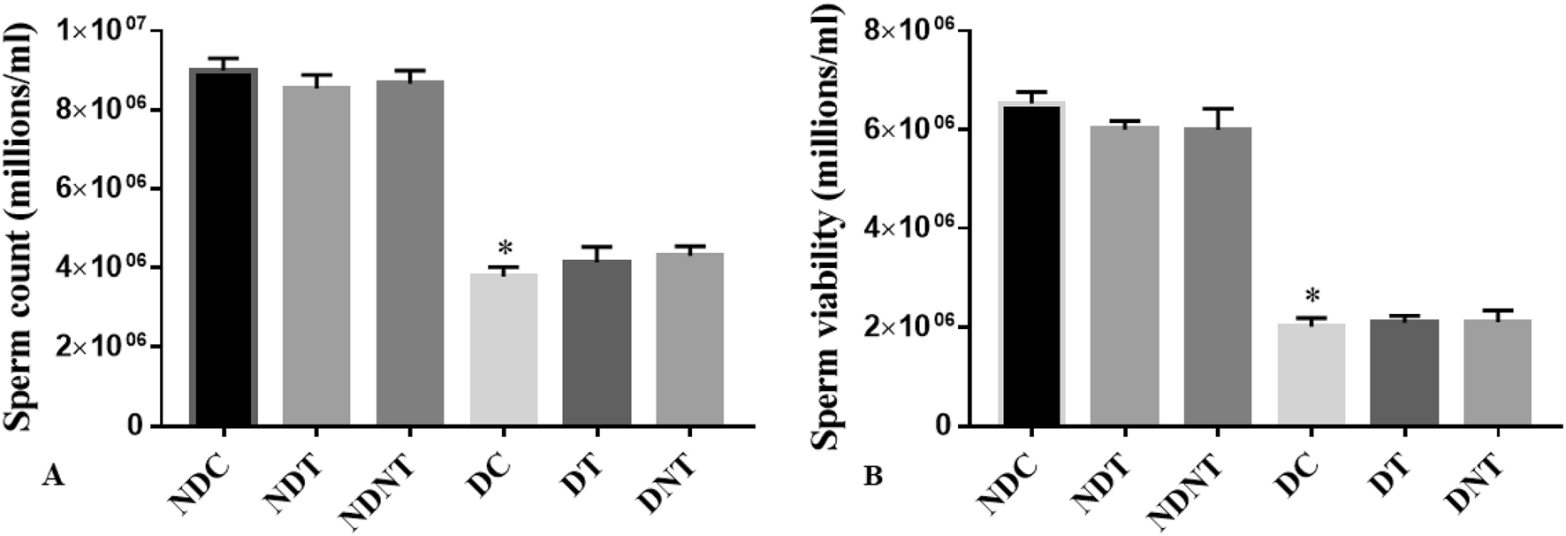
This figure describes the sperm count (**A**) and sperm viability (**B**) of the treated groups and control. As shown in (**A**,**B**), the sperm count and sperm viability in diabetic control groups were significantly reduced compared with their relative non-diabetic control groups. There were no significant differences in the sperm count and viability in DNT compared with DC.

Figure 2a shows that frt-STZ induced diabetic control (DC) rats demonstrated a significant decrease (p < 0.05) in sperm count when compared with non-diabetic control (NDC) rats. There was no significant increase in the sperm count and viability among frt-STZ induced diabetic rats treated with TDF-AgNP (DNT) compared with DC. Also, diabetic control (DC) rats showed a significant reduction in sperm viability (p < 0.05) when compared with non-diabetic control (NDC) rats (Fig. 2b).

The results are indicated as mean ± SEM of 6 animals in a group. * Compared with NDC; ^#^ vs DC. NDC-Non-diabetic control; NDT-Non-diabetic Tenofovir; NDNT-Non-diabetic nano tenofovir; DC-diabetic control; DT-Diabetic tenofovir; and DNT-Diabetic nano tenofovir. The statistical tool used was one way ANOVA followed by Turkey's multiple comparison test at p < 0.05.

### TDF-AgNP, and hormonal assay

**Figure 3:**
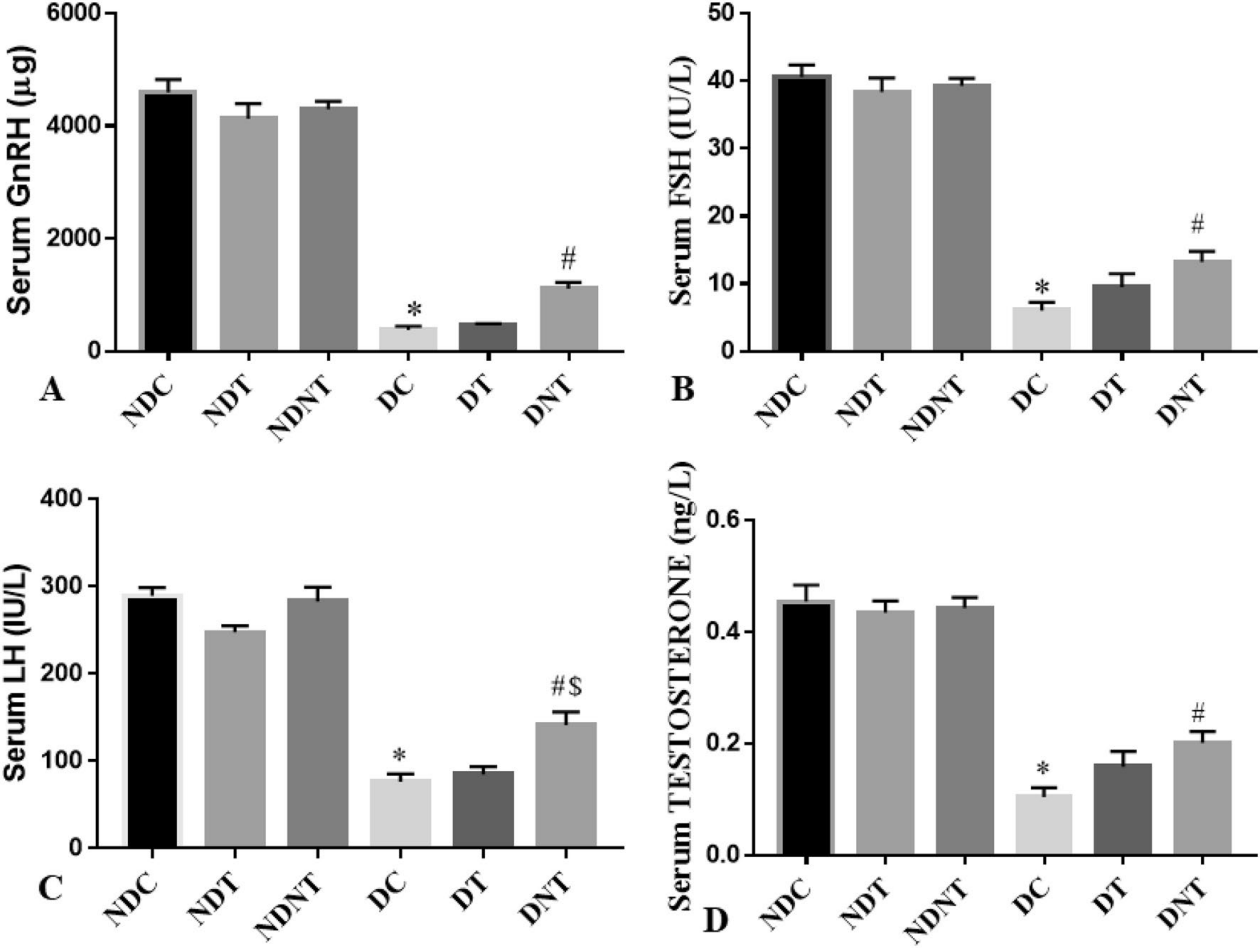
This figure demonstrates the results of the hormonal assays of the (**A**) Gonadotropin-Releasing Hormone (GnRH), (**B**) Follicle-stimulating hormone (FSH), (**C**) Luteinizing hormone (LH) and (**D**) testosterone for the treated groups and control. In (**A**–**D**), the hormonal profile (GnRH, FSH, LH, Testosterone) of the diabetic control groups were significantly reduced compared with their respective non-diabetic control groups. More so, administration of TDF-AgNP to diabetic rats significantly increases GnRH, FSH, LH and Testosterone compared with diabetic control groups.

As revealed in Fig. 3, there was a significant decrease (p < 0.05) in the level of GnRH, LH, FSH and Testosterone in the frt-STZ induced diabetic control (DC) rats compared to that of non-diabetic control (NDC) rats. However, a significant increase (p < 0.05) in GnRH, FSH and testosterone levels were observed in diabetic rats treated with TDF-AgNP compared with DC. Also, the level of LH was significantly increased (p < 0.05) in diabetic rats administered TDF-AgNP compared with DC and DT. Moreover, treatment with TDF-AgNP and TDF showed no observable effects on the hormonal profile of the non-diabetic rats (Fig. 3).

The results are indicated as mean ± SEM of 6 animals in a group. *Compared with NDC; ^#^ vs DC, ^$^ vs DT. NDC-Non-diabetic control; NDT-Non-diabetic Tenofovir; NDNT-Non-diabetic nano tenofovir; DC-diabetic control; DT-Diabetic tenofovir; and DNT-Diabetic nano tenofovir. The statistical tool used was one way ANOVA followed by Turkey's multiple comparison test at p < 0.05.

### TDF-AgNP and Insulin-like growth factor 1 (IGF-1) expression

**Figure 4:**
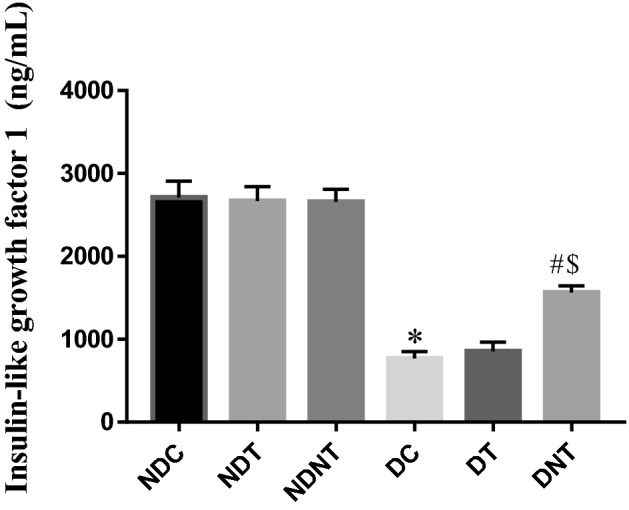
The testicular insulin-like growth factor 1 (IGF-1) of the treated groups and control. As revealed in Fig. 4, the IGF-1 level was significantly reduced in the diabetic control group compared with the non-diabetic control group. The level of testicular IGF-1 was significantly increased compared with the diabetic control group following the administration of TDF-AgNP.

In this study, there was a significant reduction (p < 0.05) in the level of IGF-1 among diabetic control rats compared to non-diabetic control (NC) rats. The IGF-1 expression in diabetic rats treated with TDF-AgNP was significantly increased (p < 0.05) compared to diabetic control (DC) rats and diabetic rats administered with TDF (DT). In addition, no significant differences were observed in the levels of IGF-1 in non-diabetic rats treated with TDF-AgNP and TDF (Fig. 4).

The results are indicated as mean ± SEM of 6 animals in a group. * Compared with NDC; ^#^ vs DC, ^$^ vs DT. NDC-Non-diabetic control; NDT-Non-diabetic Tenofovir; NDNT-Non-diabetic nano tenofovir; DC-diabetic control; DT-Diabetic tenofovir; and DNT-Diabetic nano tenofovir. The statistical tool used was one way ANOVA followed by Turkey's multiple comparison test at p < 0.05.

### Transmission electron microscopy

**Figure 5:**
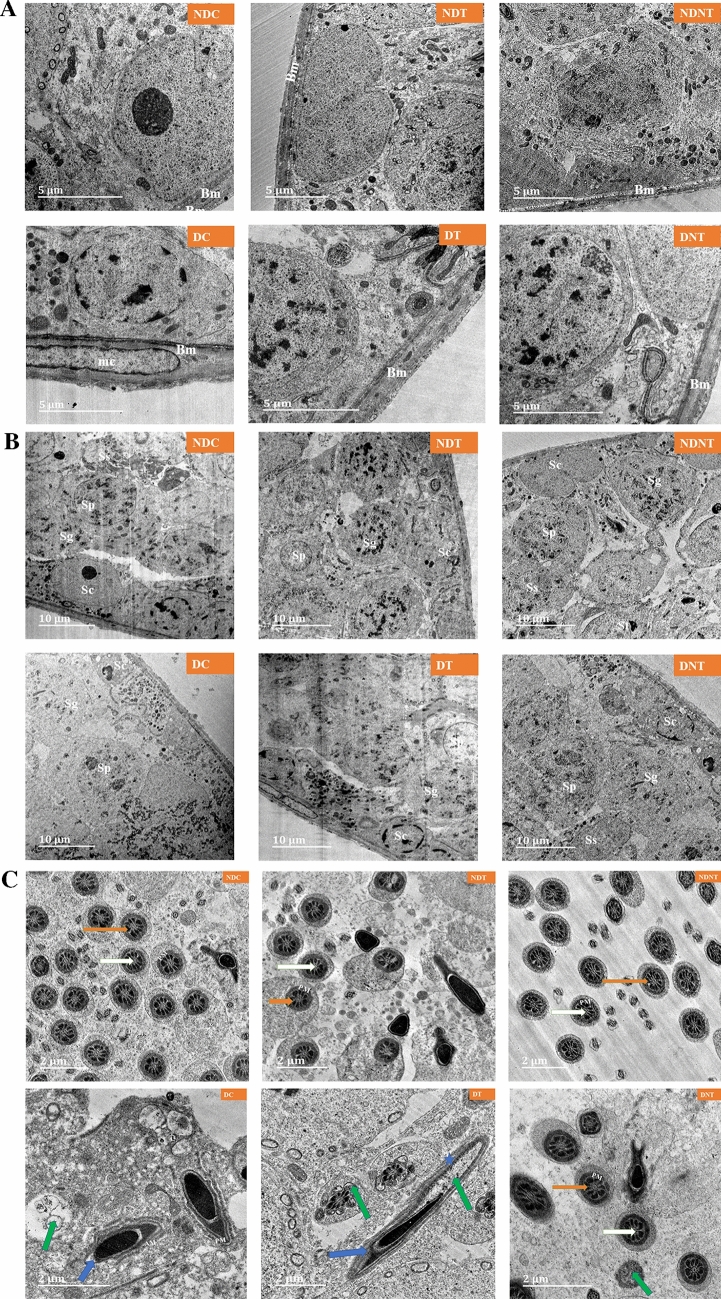
(**a**) The ultrastructure of rat testicular basement membrane across the treatment and control groups (Scale bar-5 µm). The TEM images of all the non-diabetic groups (NDC, NDT and NDNT) show well delineated basement membrane (Bm) without alterations. The ultrastructure of diabetic control rat displays thickened basement membrane and myoid cell. The basement membranes of DT and DNT groups show normal orientation. (**b**) The ultrastructure of rat testicular Sertoli and Spermatogenic cells in treated and control groups (Scale bar-10 µm). The TEM images of NDC, NDT and NDNT show a normal Sertoli cell (Sc) and Spermatogenic cells (Spermatogonia Sg, Primary spermatocyte Sp, Secondary spermatocyte Ss) in different stages of development. Ultrastructure of DC shows a degenerated Sertoli and Spermatogenic cells while DT displays degenerated spermatogonia cell. The TEM image of DNT shows an improvement in the Sertoli and Spermatogenic cells. (**c**) The ultrastructure of rat’s mature sperm middle piece across the treatment and control groups (Scale bar-5 µm). The ultrastructure of NDC, NDT and NDNT shows normal sperm axoneme characterized by central microtubules (white arrows) and nine (9) pairs peripheral microtubules encircled by the outer dense fibrils (orange arrows). Also, the concentric sperm axonemes were surrounded by the plasma membrane (PM). TEM images of DC and DT show degenerated axoneme (green arrow) nuclear membrane (blue arrow), acrosomal membrane (AM) and plasma membrane. The TEM image of DNT shows enhancement in the axonemes with little degeneration (green arrow).

The ultrastructure of NDC, NDT and NDNT shows well delineated basement membrane (Bm) without alterations. The TEM image of diabetic control rat displays thickened basement membrane and myoid cell. Also, the basement membranes of DT and DNT groups show normal orientation (Fig. 5a).

The TEM images of NDC, NDT and NDNT show a normal Sertoli cell (Sc) and spermatogenic cells; spermatogonia, primary spermatocyte, secondary spermatocyte in different stages of development. Ultrastructure of DC shows a degenerated Sertoli and Spermatogenic cells while DT displays degenerated spermatogonia cell. In contrast, the TEM image of DNT shows an improvement in the Sertoli and Spermatogenic cells (Fig. 5b).

The ultrastructure of NDC, NDT and NDNT shows well-oriented sperm axoneme characterized by central microtubules and nine (9) pairs peripheral microtubules encircled by the outer dense fibrils. Also, the concentric sperm axonemes were surrounded by the normal plasma membrane (PM). TEM images of DC and DT show degenerated axoneme, altered nuclear membrane, degenerated acrosomal membrane (AM) and deformed plasma membrane. The TEM image of DNT shows enhancement in the axonemes with little degeneration (Fig. 5c).

## Discussion

Antiretroviral drugs remain the mainstay in managing HIV-1 infection. However, the efficacy of these drugs requires long-term administration with subsequent metabolic and reproductive adverse effects. Therefore, a multi-functional drug delivery vehicle like AgNPs tends to overcome the issue of blood-testis barrier penetration and cater for metabolic and reproductive dysfunction during management of HIV infection. This study investigated the effects of TDF-AgNP conjugate on sperm quality, reproductive hormones, IGF-1 and testicular ultrastructure in diabetic rats.

Studies have shown that hyperglycaemia may alter testicular structure and function, with resultant male reproductive dysfunctions^39,40^. In this study, the elevated blood glucose level observed among diabetic rats indicated a deficiency in insulin secretion or action caused by DM induction. Previous studies have shown that the increased blood glucose level caused damage to the pancreatic β-cell with subsequent insufficient insulin secretion^41^. Interestingly, the reduced blood glucose observed in the diabetic rats treated with TDF-AgNP suggests a potent antidiabetic property of AgNP, which agrees with a similar study^42^. Previous findings showed better inhibitory activity of AgNP on the α-glucosidase, suggesting an antidiabetic effect based on the large surface area to volume ratio. This characteristic of AgNP increases surface area for electron transfer reaction and increase pharmacokinetics^43^.

It has been reported that testicular dysfunction characterized by reduced sperm motility, sperm count, sperm viability, and seminal fluid quantity is frequently observed in diabetic patients compared to their non-diabetic counterparts^40^. The significant decrease in sperm count and viability in diabetic control rats in this study suggests the deleterious effects of diabetes on indices of testicular physiology. This finding concurs with Ding and colleagues, that described alterations in sperm quality, seminal fluid, sperm motility and sperm DNA integrity as consequences of diabetes mellitus^44^. More so, sperm counts, and viability of diabetic rats treated with TDF-AgNP showed no significant changes compared with diabetic control indicating that TDF-AgNP did not affect the sperm count and viability in diabetic rats. In addition, treatment with TDF-AgNP and TDF caused no alterations in sperm count and viability in non-diabetic rats. These observations may be due to the size, shape, and properties of silver nanoparticles used in this present investigation.

The hypothalamic-pituitary–gonadal axis (HPGA) is a complex system that involves the endocrine control of testicular function. In this axis, the hypothalamus releases gonadotropin-releasing hormone (GnRH), which in turn causes the pituitary gland to secrete follicle-stimulating hormone (FSH) and luteinizing hormone (LH). Similarly, LH stimulates the Leydig cells of interstitial testicular tissue to produce testosterone. The secreted testosterone and FSH control the Sertoli cells responsible for spermatogenesis^45,46^. Therefore, any imbalance in this axis or the component impairs testicular functions. In this study, a significant decrease in GnRH, LH, FSH, and testosterone recorded in diabetic control rats suggest that DM disrupted the HPGA with potential testicular toxicity. Schoeller et al. (2012) attributed a reduction in insulin levels to an effect of hyperglycaemia, which leads to decreased leptin levels and, subsequently, a decrease in GnRH secretion. Consequently, a decrease in GnRH secretion causes a reduction in FSH and LH secretions, altering the testicular functions^47^.

The significant increase in the serum levels of GnRH, FSH, LH, and testosterone observed in the diabetic rats treated with TDF-AgNP in this investigation may be attributed to the impact of AgNP to increase serum insulin, ultimately leptin level. A previous study examined the antidiabetic activities of zinc oxide nanoparticles and AgNPs in diabetic rats. Their findings demonstrated the ability of AgNP to reduce blood glucose levels, increase serum insulin, increase glucokinase activity and elevate the expression of insulin and insulin receptors^42^. Since an increase in insulin levels leads to a rise in leptin levels and a subsequent increase in GnRH secretion, hence, increase in the hormonal profile obtained in diabetic rats treated with TDF-AgNP may be explained by this mechanism. More so, treatment with TDF-AgNP and TDF caused no reduction in these hormones among non-diabetic rats, suggesting that the TDF-AgNP and TDF did not alter the hormonal profiles in non-diabetic rats.

Proper functions of the testis rely on the multiple interactions between endocrine and exocrine glands through the hypothalamic-pituitary–gonadal axis. In addition, IGF, the internal gonadal constituents, control reproductive function and development^15,48^. The role of IGF-1, a family of IGF, in supplying vital signals that mediate the reproductive functions, metabolism and growth, have been detailed^15,49^. Also, disruption in the IGF signalling pathways has been implicated in metabolic disorders, RDs, DM, dementia, cancer, and decreased life expectancy^50,51^.

In this present study, the significant reduction in testicular IGF-1 in diabetic control rats suggest a disruption in the IGF-1 axis with a subsequent harmful effect on reproductive function. Studies have reported the correlation between IGF-1 signalling system alteration and reproductive dysfunction. These reports attributed insulin deficiency to the considerable reduction in the concentration of testicular IGF-1 noticed in diabetic rats^52,53^.

Furthermore, a significant increase in IGF-1 concentration in diabetic rats treated with TDF-AgNP may be due to the ability of AgNP to increase insulin levels. This postulation is based on the fact that AgNP reduces blood glucose levels^42^ and improves insulin sensitivity and serum insulin with a resultant increase in the concentration of IGF-1. Alkaladi and colleagues have documented a significant increase in the serum insulin concentration among diabetic rats administered AgNPs, which further supports the present study's result. More so, AgNP has a bioactive compound that inhibits alpha-amylase and alpha-glucosidase, essential enzymes in carbohydrate metabolism^42^. The inhibition of these enzymes causes a reduction in blood glucose levels. In addition, IGF-1 lowered blood glucose, ensured glycaemic control and improved insulin sensitivity, as demonstrated by the previous investigation^54^. Since TDF-AgNP increases IGF-1 concentration in diabetic rats, indicating that it may restore the function of HPGA, normalize the hormonal profile and enhance testicular function in diabetic conditions.

The TEM examination serves as a tool to appreciate the cellular architecture and understand the testicular physiology and the status of the spermatozoa. Also, it aids a better understanding of the cellular organelles and their functions in reproduction^55^. More so, TEM plays a role in identifying abnormalities in the morphology and the functions of the testicular tissue. The basement membrane represents one of the essential architectural components of the testis, which can be examined ultra-structurally. The testicular basement membrane maintains the testis structural integrity and functional components, and alteration in the basement membrane has been implicated in various reproductive derangements^56^. In this study, architectural alterations such as a thickened basement membrane, and myoid cells, as well as degenerated Sertoli cells, spermatogenic cells, acrosomal membrane, nuclear membrane, plasma membrane and axoneme alteration observed in diabetic control rats suggested an impairment in the testicular structural and functional integrity. This finding conforms with the previous study that reported thickened basement membrane, degenerated germ cells, and reduced seminiferous tubule diameter in diabetic rats^57^. These derangements were delineated as morphological markers of reproductive dysfunction through oxidative stress and apoptotic mechanisms^58,59^.

Previously, free radical-induced oxidative stress has been described as the mechanism involved in testicular damage by weakening the antioxidant defence system and causing chronic inflammation^60–62^. Moreover, animal experiments have shown that AgNP possesses anti-inflammatory properties^63^ and the ability to scavenge free radicals^64^. These properties thereby improve the testicular antioxidant system, reduce the free radical-induced inflammatory process and restore the structural and functional integrity of the testis. In this study, few axoneme alterations noticed in the diabetic rats treated with TDF-AgNP indicated an improvement in the testicular architecture previously marked with various degrees of distortions in diabetic control rats. This improvement may be attributed to the antioxidant and anti-inflammatory activities of AgNP.

## Conclusion

This present study has revealed that administration of TDF-AgNP to diabetic rats neither affects sperm count nor sperm viability. More so, diabetic rats treated with TDF-AgNP showed a significant increase in GnRH, FSH, LH and testosterone, and IGF-1 compared to their respective diabetic control. Also, administration of TDF-AgNP to diabetic rats improved the testicular architecture marked with a thickened basement membrane, degenerated and irregular Sertoli cells, disorganized spermatogenic cells and degenerated axoneme. In addition, administration of TDF-AgNP significantly reduced blood glucose levels in diabetic rats. Ultimately, this study has shown that TDF-AgNP increases IGF-1 concentration, reduces blood glucose levels in diabetic rats, restores the function of HPGA, normalizes the hormonal profile, and enhances testicular function in diabetic rats. Further investigation into the interactions of this nano-drug on the testicular parameters at the molecular level is thereby required.

## Data Availability

The data that supports this study are available in the methods and results sections.

## Abbreviations

RDs: Reproductive dysfunctions
HIV: Human immunodeficiency virus
ARVDs: Antiretroviral drugs
TDF: Tenofovir disoproxil fumarate
AgNPs: Silver nanoparticles
TDF: AgNPs-Tenofovir disoproxil fumarate loaded silver nanoparticle
IGF-1: Insulin-like growth factor 1
T2DM: Type 2 diabetes mellitus
Frt: Fructose
STZ: Streptozotocin
Frt-STZ: Fructose-streptozotocin
GnRH: Gonadotropin-releasing hormone
FSH: Follicle stimulating hormone
LH: Luteinizing hormone
HAART: Highly active antiretroviral therapy
NPs: Nanoparticles
DM: Diabetes Mellitus
PLGA: Poly (lactic-co-glycolic acid)
UV–Vis: Ultraviolet visible spectroscopy
FTIR: Fourier transform infrared spectroscopy
HR-TEM: High-resolution transmission electron microscope
FESEM: Field emission scanning electron microscope
EDX: Energy dispersive X-ray
ELISA: Enzyme-linked immunosorbent assay
HPGA: Hypothalamic-pituitary–gonadal axis
TEM: Transmission electron microscopy
